# *Nosema ceranae* Infection Promotes Proliferation of Yeasts in Honey Bee Intestines

**DOI:** 10.1371/journal.pone.0164477

**Published:** 2016-10-13

**Authors:** Aneta A. Ptaszyńska, Jerzy Paleolog, Grzegorz Borsuk

**Affiliations:** 1 Department of Botany and Mycology, Institute of Biology and Biochemistry, Faculty of Biology and Biotechnology, Maria Curie-Skłodowska University, Akademicka 19, 20–033 Lublin, Poland; 2 Department of Zoology, Ecology and Wildlife Management, University of Life Sciences in Lublin, Akademicka 13, 20–950 Lublin, Poland; 3 Department of Biological Basis of Animal Production, Faculty of Biology and Animal Breeding, University of Life Sciences in Lublin, Akademicka 13, 20–950 Lublin, Poland; Arizona State University, UNITED STATES

## Abstract

**Background:**

*Nosema ceranae* infection not only damages honey bee (*Apis melifera*) intestines, but we believe it may also affect intestinal yeast development and its seasonal pattern. In order to check our hypothesis, infection intensity versus intestinal yeast colony forming units (CFU) both in field and cage experiments were studied.

**Methods/Findings:**

Field tests were carried out from March to October in 2014 and 2015. *N*. *ceranae* infection intensity decreased more than 100 times from 7.6 x 10^8^ in March to 5.8 x 10^6^ in October 2014. A similar tendency was observed in 2015. Therefore, in the European eastern limit of its range, *N*. *ceranae* infection intensity showed seasonality (spring peak and subsequent decline in the summer and fall), however, with an additional mid-summer peak that had not been recorded in other studies. Due to seasonal changes in the *N*. *ceranae* infection intensity observed in honey bee colonies, we recommend performing studies on new therapeutics during two consecutive years, including colony overwintering. A natural decrease in *N*. *ceranae* spore numbers observed from March to October might be misinterpreted as an effect of *Nosema* spp. treatment with new compounds. A similar seasonal pattern was observed for intestinal yeast population size in field experiments. Furthermore, cage experiments confirmed the size of intestinal yeast population to increase markedly together with the increase in the *N*. *ceranae* infection intensity. Yeast CFUs amounted to respectively 2,025 (CV = 13.04) and 11,150 (CV = 14.06) in uninfected and *N*. *ceranae*-infected workers at the end of cage experiments. Therefore, honey bee infection with *N*. *ceranae* supported additional opportunistic yeast infections, which may have resulted in faster colony depopulations.

## Introduction

A significant decrease in the number and biodiversity of pollinators has been observed over the last 50 years. It entails huge economic losses and has even been called a “pollination crisis” [1]. A decline in honey bee populations is caused by combined stress from parasites, pesticides, and bees’ diet being inadequate due to reduced diversity of wildflower resources, which are replaced by monocultures [2]. Consequently, weakened honey bees are more easily attacked by many diseases, *inter alia* those caused by fungi such as *Nosema apis* and *Nosema ceranae* belonging to the Microsporidia phylum, Balbiani 1882 [3].

Although these two fungal species inhabit the same host i.e. *Apis mellifera*, and have similar life cycles, a phylogenetic analysis of multiple sequence data sets indicated that they are not closely related. The analysis revealed that *N*. *ceranae* is a sister species to *N*. *bombi*, whereas the weight of evidence is consistent that *N*. *apis* is a basal member of the *Nosema* clade [4, 5]. Competition between these two parasites within the same host leads to conflicts that may influence parasitic virulence evolution as can be seen in the differences in seasonal variations and gross colony infection intensity symptoms described for *N*. *apis* and *N*. *ceranae* [5, 6, 7, 8]. Infection of *A*. *mellifera* with *N*. *apis* was well described over 100 years ago [9, 10] whereas it was not until 2005 that the first infection of *A*. *mellifera* with *N*. *ceranae* was recorded in Taiwan [11]. Soon after that *N*. *ceranae* spread to Europe and both Americas, where it has become prevalent [6]. Therefore, *A*. *mellifera* and *N*. *apis* make a mutually co-adapted endemic host-pathogen pair that is subject to invasion of a new pathogen, i.e. *N*. *ceranae* in the temperate climate zone. This is well reflected in the way the honey bee immune system responds to these parasites, since *N*. *apis* infection increases immune defense while *N*. *ceranae* leads to immunosuppression in *A*. *mellifera* [12]. Presently, a wide spread of *N*. *ceranae* is responsible for numerous honey bee infections in Europe [13, 14] and is believed to be displacing *N*. *apis* all over the world [5, 15, 16, 17, 18]. We hypothesized that such an invasion by a new pathogen developing in apian intestines [19] may affect development of other intestinal microbiota. We initially confirmed these suspicions during our preliminary research into honey bee intestinal yeast populations [20]. In previous cage experiments performed throughout two summer months, an interdependence between *N*. *ceranae* infection intensity and intestinal yeast development was observed, albeit merely in four measurements, each completed at the end of a single experiment [20]. Cage data may differ from those obtained in fully functional colonies [21, 22, 23] and seasonal changes are crucial for understanding interrelations between *N*. *ceranae* and yeasts within apian intestine microenvironment. Hence, there is a need for field research into the impact of *N*. *ceranae* on *N*. *apis* and honey bees as well as honey bee intestinal yeast populations. Not only is it significant for the protection of honey bees, but also for broadening the knowledge concerning inter-pathogen relations occurring during new pathogen invasions.

*N*. *ceranae*, just like *N*. *apis*, completes its life cycle in honey bee intestines. Even a medium *Nosema* spp. infection causes a complete overlay of the gut with spores, which disturbs absorption of nutrients [24]. Moreover, *Nosema* spp. infection affects natural honey bee intestinal microbiota, which include about: 70% of Gram-negative bacteria mainly from the Enterobacteriaceae, Alcaligenaceae and Pseudomonadaceae families, 27% of Gram-positive bacteria, primarily from the genus *Bacillus* and less than 1% of yeasts and other fungi, *inter alia Saccharomyces rouxi*, *S*. *mellis*, *S*. *bisporus*, *S*. *roesi*, *S*. *bailli*, *S*. *heterogenicus*, *Aspergillus* sp., *Alternaria* sp., *Cladosporium* sp., *Penicillium* sp., *Pichia* (*Hansenula*) *anomala*, *Rhizopus arrhizus*, *Torulopsis* sp. [25, 26, 27, 28, 29, 30, 31, 32]. However, it must be highlighted that yeasts can be treated as honey bee stress indicators, because healthy honey bees do not have intestinal yeasts or have them very little [20, 25, 26, 27]. Furthermore, adding yeasts to honey bee diet caused damage to the midgut epithelial layer, which drastically disturbed the absorption of nutrients and significantly shortened bees’ lifespans [33, 34, 35, 36, 37]. Therefore, yeasts are most likely honey bee opportunistic pathogens whose population expands during disruption of homeostasis in their host. Our preliminary study on caged honey bees [20] suggested that intensity of *Nosema* infection influenced honey bee intestinal yeast population size in two ways. A slight infection increased the yeast number, whereas a heavy infection reduced it. We wondered, however, if such interrelationships would be observed on a colony level as well, and whether they would vary reflecting seasonal changes of *Nosema* spp. population sizes. An infection intensity increase was recorded in the spring, which was followed by a marked decline in the summer and fall (*inter alia*: [38, 39, 40, 41]). On the other hand, *N*. *ceranae*, which develops in the honey bee intestine, ought to compete for attachment sites and nutrients with the remaining pathogenic fungi, including *N*. *apis* and yeasts. Therefore, the question arises whether a significant increase in the intestinal yeast population could finally suppress *Nosema* spp. infection intensity, and *vice versa* on the colony level. Moreover, we wondered if seasonal changes in *Nosema* intensity could be detected through cage tests performed parallel to field studies.

The aim of this research was to study how the seasonal pattern of the *N*. *ceranae* infection intensity influenced development of intestinal yeast population in honey bees from March to October through two consecutive years, based on both cage and field assays.

## Material and Methods

This research was supported by an Individual Research Grant from the Vice-rector for Research and International Relations of UMCS (Lublin, Poland) for Aneta A. Ptaszyńska. The funder had no role in the study design, data collection and analysis, decision to publish, or preparation of the manuscript.

Our research had been planned in a way that reduced the number of honeybees to the minimum necessary for the proper execution of these experiments and had been accepted by the head of the Department of Experimental and Environmental Biology, University of Life Sciences in Lublin (Poland). Afterwards, honey bees, *Apis mellifera carnica*, were collected from the university apiary of the University of Life Sciences in Lublin.

Field and cage tests were carried out at the apiary of the University of Life Sciences, Lublin, Poland (51°13'32.2"N 22°38'08.3"E) and the laboratory of the Maria Curie-Skłodowska University, Lublin, Poland (51°14'35.2"N 22°32'33.3"E), respectively.

### 2.1. The field test protocol

Hive bottom-drawers were placed individually in 82 *A*. *mellifera carnica* colonies at the beginning of the overwintering period 2013–2014 in order to select colonies for field tests. Dead worker-bees were sampled from winter falls. They were collected separately from each colony (drawer) in early February 2014. Next, 50 workers from each colony sample were homogenized and examined for the presence of *Nosema* spp. spores (section 2.4). Colonies in which no spores were found were considered uninfected. Whenever *Nosema* spp. spores were found, the colony was classified as infected and put in a secluded place. No nosemosis treatment was administered. Further DNA analysis (section 2.3) revealed which colony was infected with *N*. *apis*, which with *N*. *ceranae*, and which with both these pathogens (mixed infection). Consequently, a colony infection intensity, defined as the number of *N*. *ceranae* spores observed in a single host intestinal environment [42], was assessed through determining the number of spores in the 50-worker colony homogenate, whereas the infection type was specified by means of a DNA analysis.

At the end of February 2014, three colonies infected solely with *N*. *ceranae* at a similar intensity and three colonies considered uninfected were qualified to obtain biological material for studying the seasonality of the *N*. *ceranae* infection course and its impact on the intestinal yeast CFU number. Then, in late February 2015, colonies that had been classified as infected in 2014 were checked again to determine the intensity and type of infection. Two of them, which showed similar intensity of infection with *N*. *ceranae* and also two uninfected colonies were qualified for a repetition of the study.

100 live worker-bees were sampled from each selected colony in both 2014 (6 colonies) and 2015 (4 colonies) during the first five days of each month from March to October. Each 100-bee colony sample was divided into units of 10 worker-bees to check the *Nosema* infection type on the basis of DNA analysis (section 2.3), to establish *N*. *ceranae* intensity (in triplicate, section 2.4), and to determine yeast CFU number (in triplicate, section 2.5). The remaining 30 workers from every 100-bee colony sample were treated as a reserve. Therefore, the database to establish the *N*. *ceranae* intensity was: 3 pooled samples of 10 bees taken on a monthly basis from each of the 6 (2014) or 4 (2015) colonies during 8 months, i.e. 144 samples of 10 bees in 2014 and 96 samples of 10 bees in 2015. The same number of samples was taken for intestinal yeast CFU analysis.

### 2.2. The cage test protocol

Two consecutive cage experiments (repetitions) were additionally conducted, one in June and one in July, both in 2014 and 2015 (four experiments in total) applying the following protocol: the sealed-brood combs on the 20^th^ day of the brood development were taken out from a *Nosema*-free source colony and incubated in an air-conditioned chamber (35°C, RH = 60%). Next, freshly emerged workers were placed in 80 wooden cages, 40 workers per cage, and kept under laboratory conditions (darkness; 30°C; RH = 65%). The first worker sample, comprising 40 workers (2 x 10 workers for DNA analysis and 2 x 10 workers for yeast cultures) was randomly taken from all the cages one day after the workers’ caging. Molecular analysis (section 2.3) proved there was no *N*. *apis* and *N*. *ceranae* DNA in the sampled workers. Intestinal yeasts were cultured as described in section 2.5. On the third day after worker emergence, the cages were divided into two groups, 40 cages in each. In the first group, workers were infected with *N*. *ceranae*, according to the methodology described by Forsgren and Fries [43], whereas in the second, they were uninfected and maintained as a control. The cages with control (uninfected) and *N*. *ceranae* infected honey bee workers were kept in separate chambers (darkness; 30°C; RH = 65%) to prevent spore contamination.

Then, beginning from the second day after the infection of workers (4-day-old workers), the samples of 40 workers, were taken every day for 18 days (2 x 10 workers for *N*. *ceranae* spore counting, section 2.4 and 2 x 10 workers for yeast cultures, section 2.5).

Summing up, 2 groups x 18 samplings = 36 samples of 10 workers to count *N*. *ceranae* spores and the same number of samples to culture intestinal yeasts, were taken both in 2014 and 2015.

### 2.3. Total DNA extraction and detection of *N*. *apis* and *N*. *ceranae* using duplex PCR

DNA was extracted from each pooled sample of 10 honey bee workers, which were ground in sterile water. 100 μL of each homogenate was added to 180 μL of lysis buffer and 20 μL of proteinase K, and the total DNA was isolated with the DNeasy Blood and Tissue Kit (Qiagen) according to the producer’s instruction. Every isolate was used as a template for detection of *N*. *apis* and *N*. *ceranae* specific 16S rDNA by PCR with *Nosema*-specific primers: 321-APIS for *N*. *apis* and 218-MITOC for *N*. *ceranae* as described in Martin-Hernandez *et al*. [44].

### 2.4. *Nosema* spp. infection intensity

Abdomens were individually dissected from the workers and homogenated by grinding in a ratio of 1 abdomen for 1 mL of sterile water. 50 workers in case of field tests (section 2.1), and 10 workers in case of the cage test (section 2.2) were used to prepare one homogenate. After that, the homogenates were smeared on a microscope slide for examination. For each spore suspension, averages of 2 intensity estimates were used. The number of *Nosema* spp. spores per honey bee worker was estimated using Olympus BX 61 light microscope and a haemocytometer [6, 45].

### 2.5. Estimation of yeast colony forming units (CFUs)

Ten workers from each pooled sample, both from the field (section 2.1) and from the cage (section 2.2) experiments were individually surface sterilized in 70% ethanol and delicately (aseptic conditions) pressed to discharge feces into one sterile Eppendorf tube, 10 workers per one tube. The feces collected in each tube were mixed to obtain homogenized solution. After that, 150 μL of each homogenate was suspended in 150 μL of sterile 0.6% NaCl invertebrate saline to obtain 300 μL of the feces suspension. Then, two samples, per 100 μL of the suspension, were immediately spread in duplicate on Petri dishes containing Sabouraud dextrose agar with chloramphenicol and gentamycin. Furthermore, 100 μL of 10^−2^- and 10^−4^-fold dilutions of the preliminary feces solution were also cultivated in duplicate on Petri dishes. Each Petri dish, was incubated for 5 days after the feces spreading at 30°C. The addition of antibiotics inhibited bacteria growth and improved the cultivation of microfungi. The API® strips-Yeasts (bio Mérieux Clinical Diagnostics) were used to differentiate fungi isolated from honey bee workers’ intestinal tracts and found on the Petri dishes.

A separate procedure was performed in order to estimate the number of yeast colony forming units (CFU) per one honey bee worker. 50 honey bee workers were individually weighed and then pressed to discharge all feces into separate small tubes, one tube per worker. The volume and weight of feces discharged per worker were measured. The average honey bee worker weight was 155.7 mg, while the average weight of feces discharged per one worker was 29 mg. The average volume of single-bee feces was 23 μL. Therefore, it was assumed that 150 μL of the feces-homogenate, and consequently 300 μL of the feces solution in 0.6% NaCl saline originated on average from about 6.5 worker honey bees. Consequently, it is assumed that feces from 2.17 workers were spread on one Petri dish, when 100 μL of the feces solution was used.

### 2.6. Statistical analysis

All statistical analyses were performed using SAS software [46]. One-way ANOVA (*N*. ceranae-infected *versus* uninfected), in which a group effect was the experimental factor, and Student's t test were applied to compare differences between groups.

**Fig 1:**
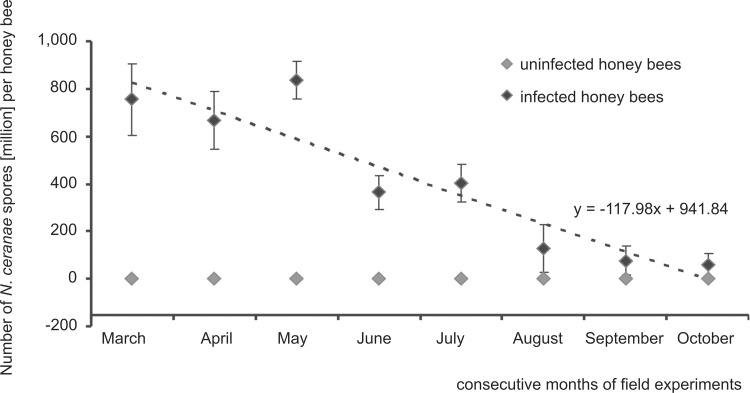
Field tests. **Seasonality in the *N*. *ceranae* infection intensity observed from March to October in naturally infected honey bee colonies.** Data obtained from measurements conducted on a monthly basis for the first five days of each month from March to October in 2014 and 2015. The dotted line represents a linear regression. Diamond shapes (♦) represent means while error bars indicate standard deviation.

To estimate dependency between yeast CFU number and *N*. *ceranae* spore number, Model I linear regression for field tests and Spearman’s correlation for cage tests were used. Linear regression, however, does not evaluate significances of deviations from the general trend (discrepancy between the real data and regression line), i.e. does not answer whether a given peak (e.g. Fig 1) is a statistical (random) artifact or a significant deviation from the general trend. Therefore, to assess a relationship between the numbers of spores (infection intensity) along subsequent months (seasonality) the regression model with dummy variables (month indicators) was also applied (Model II). Model parameters were estimated using the Gauss-Newton least squares method [47].

## Results

### 3.1. Field tests

An analysis of the winter debris from 82 colonies, which was performed in 2014, revealed that 11 of them were *Nosema* infected (Table 1). Only one of these 11 colonies was infected solely with *N*. *apis* while as many as six were infected solely with *N*. *ceranae*. Intensity and infection type were similar in 2014 and 2015. However, the colony infected solely with *N*. *apis* in 2014 was found in 2015 to be infected both with *N*. *apis* and *N*. *ceranae*. Due to the lack of nosemosis treatment, an increase in the *N*. *ceranae* infection intensity was clearly observed in 8 out of 11 colonies during the second year of our studies. No live bees were found in the two most intensively infected colonies. On the other hand, one colony infected at a very low intensity in 2014 was found uninfected next year.

**Table 1:** Infection types and intensities in the *Nosema* spp. infected colonies.

Colony no.		1	2	3	4	5	6	7	8	9	10	11
II 2014	*N*. *apis*	**+**	**+**	**+**	**+**	**+**	**-**	**-**	**-**	**-**	**-**	**-**
	*N*. *ceranae*	**-**	**+**	**+**	**+**	**+**	**+**	**+**	**+**	**+**	**+**	**+**
	number of spores [million]	244	1,160	940	624	488	1,800	**828**	**792**	**664**	320	0.8
II 2015	*N*. *apis*	+	+	+	+	+	**-**	**-**	**-**	**-**	**-**	**-**
	*N*. *ceranae*	+	+	+	+	+	+	+	+	+	+	+
	number of spores [million]	328	Θ1,480	820	780	560	Θ1,920	**984**	**920**	592	640	0.0

(+ or -); a given infection type was found or not found. The colonies analyzed for the monthly *N*. *ceranae* number of spores and yeast CFU number are **in bold.** The colonies depopulated in 2015 are marked with Θ.

Monitoring of the *N*. *ceranae*-infected colonies for 8 months showed that average number of spores decreased more than 100 times from May to October both in 2014 and 2015 (Fig 1). In 2014, the decrease was from approximately 7.6 x 10^8^ per honey bee worker in March to 5.8 x 10^6^ in October, whereas in 2015 the spore number decreased from 9.5 x 10^8^ to 7.1 x 10^6^, respectively. Despite this general trend to reduce the number of spores from spring to fall, which was significant (p < 0.05) in both regression Model I and Model II, two peaks were observed. Both peaks were considered to be temporary rises in *Nosema* infection intensity, one in May and the other in July. Corrections for the linear trend of May and July (Model II) were significant (p < 0.05). Additional calculations for Model II indicators were performed taking into account only periods when biggest increases in the number of spores were observed. In both such periods discrepancies from the linear trend were significant (p < 0.05). This indicates that the peaks were not statistical artefacts.

**Fig 2:**
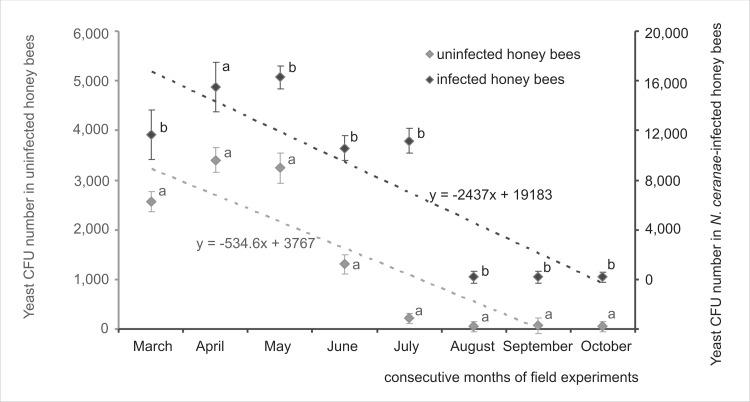
Field tests. **Changes in the intestinal yeast CFU number per honey bee worker observed from March to October in uninfected and *N*. *ceranae*-infected honey bee colonies.** Data obtained from measurements conducted on a monthly basis for the first five days of each month from March to October in 2014 and 2015. Different lower case letters indicate significant differences (p < 0.05) between uninfected and *N*. *ceranae*-infected colonies (one-way ANOVA was performed separately for each month). Dotted lines represent regression lines. Diamond shapes (♦) represent means while error bars indicate standard deviation.

Fungi belonging to *Candida* and *Saccharomyces* genera were detected both in *N*. *ceranae*-infected and uninfected colonies. The number of intestinal yeast CFUs isolated both from uninfected and the *N*. *ceranae*-infected colonies decreased from May to October in 2014 and 2015 but CFU number was always markedly higher in infected colonies (Fig 2) (one-way ANOVA p < 0.05 was performed separately for each month). General seasonal patterns of changes in intestinal yeast CFU, showing one peak in May and later a decrease were similar in infected and uninfected colonies. However, *N*. *ceranae*-infected colonies had an additional summer peak in July. Both CFU peaks, i.e. those observed in May and July, corresponded with *N*. *ceranae* infection intensity peaks (compare Figs 1 and 2). Generally, the yeast CFU number was positively correlated with the *N*. *ceranae* infection intensity (Table 2), particularly in spring and early summer.

**Table 2:** Field tests. Spearman’s correlation coefficients between yeast CFU and *N*. *ceranae* number of spores. Significant correlations (p < 0.05) are indicated with an asterisk.

*Nosema ceranae* number of spores		
Yeast CFU number	March	0.6686*
		p = 0.025
	April	0.6393*
		p = 0.034
	May	0.3624
		p = 0.273
	June	0.7114*
		p = 0.014
	July	0.2312
		p = 0.494
	August	-0.4737
		p = 0.141
	September	-0.5528
		p = 0.078
	October	0.3007
		p = 0.369

### 3.2. Cage experiments

**Fig 3:**
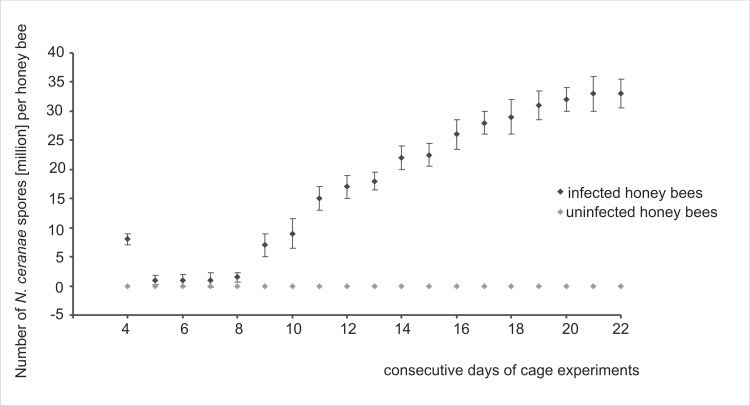
Cage tests. **The average *N*. *ceranae* infection intensity observed during consecutive days of cage experiments conducted in 2014 and 2015.** Data obtained from daily sampling from the second day after the infection (4-day-old workers) to the end of each experiment in 2014 and 2015. Diamond shapes (♦) represent means while error bars indicate standard deviation.

Honey bee workers were artificially infected with *N*. *ceranae* on the third day after their emergence. *Nosema* spores swallowed by honey bee workers were observed in microscopic samples shortly after the infection. On the second day and on the third day after the infection, spore numbers dramatically dropped (Fig 3). There were so few spores in some samples that their number was indeterminable. After that, a slight increase in the *N*. *ceranae* infection intensity was observed, and from the 8^th^ day it became markedly intensive and permanent.

**Fig 4:**
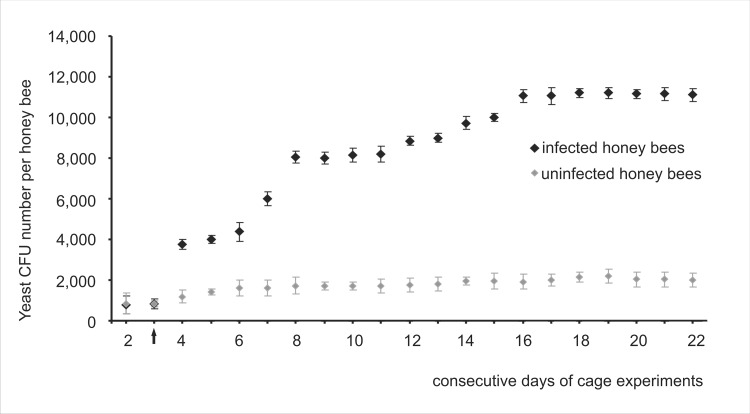
Cage tests. **The average yeast CFU number observed during consecutive days of cage experiments conducted in 2014 and 2015.** Data obtained from daily sampling from the second day of workers emerging to the end of each experiment in 2014 and 2015. The arrow (↑**)** indicates the day of *N*. *ceranae* infection. Diamond shapes (♦) represent means while error bars indicate standard deviation.

Similarly to field tests, fungi belonging to *Candida* and *Saccharomyces* genera were detected in feces of honey bees kept in cages. In uninfected honey bee workers, intestinal yeast CFU number grew slightly during the experiments (Fig 4), from 845 CFUs (CV = 24.32) detected on the second day, to 2,025 CFUs (CV = 13.04) at the end of the investigations (Fig 4). In *N*. *ceranae*-infected workers, the growth was markedly higher and two peaks were observed; the first on the 4^th^ and the second on the 8^th^ day of experiments. Both peaks were connected with the *N*. *ceranae* infection intensity. The first was observed a day after the bees had been infected and the second was connected with a substantial increase in *N*. *ceranae* spore number. Therefore, cage experiments confirmed that the size of the honey bee intestinal yeast population markedly increased after bees were infected with *N*. *ceranae* and was connected with the infection intensity (Fig 4). It was also confirmed with Spearman’s correlation between numbers of yeast CFUs and *N*. *ceranae* spores, whereas the correlation coefficient estimated in cage experiments was 0.82 (p = 0.0121) in 2014 and 0.79 (p = 0.0119) in 2015.

Generally, it was in the *N*. *ceranae*-infected honey bee workers that the intestinal yeast CFU number reached the highest values both during field and cage tests. A comparison of cage and field tests indicated that more intestinal yeast CFUs were found in bees sampled from colonies (field experiments). Yeast CFU amounted to 11,200 (CV = 13.44) on the 19^th^ day of the experiment in *N*. *ceranae*-infected caged workers whereas 16,280 CFU (CV = 14.16) were detected in May in *N*. *ceranae*-infected colony workers.

## Discussion

Only one colony was found to be infected solely with *N*. *apis* out of 11 *Nosema* spp. infected colonies that were detected from the pool of 82 honey bee colonies monitored in 2014. Next year, however, the colony was found already as mix-infected, i.e. infected both with *N*. *apis* and *N*. *ceranae* (Table 1). Therefore, the *N*. *ceranae* infections were markedly prevalent. Probably, it was a weak immune response of *A*. *mellifera* to the *N*. *ceranae* infection that was the reason for this situation, since *N*. *ceranae* caused immunosuppression in honey bees [12]. Surprisingly, we noticed that one of the colonies infected with *N*. *ceranae* at a low intensity (0.8 million spores per honey bee) in 2014 was found self-cured (uninfected) next year, despite the fact that nosemosis was not treated. This may indicate that there are some defense mechanisms against *N*. *ceranae* in honey bees. If this phenomenon is confirmed, new possibilities for resistant honey bee breeding may arise.

Until recently, *N*. *ceranae* dominated in warmer climates showing non-seasonal nosemosis course [18, 44, 48]. At present, *N*. *ceranae* infections spread through temperate climate zones and were detected even in the severe climate of Finland [17, 43, 44], nonetheless having a seasonal course there. In our studies, a pattern of *Nosema ceranae* infections was similar to that described for temperate zones of USA, Canada, and Germany [38, 39, 40, 41], showing a spring peak and subsequent decline in the summer and fall (Fig 1). However, in our study, the second peak was observed in the middle of the summer in two consecutive years. It is believed that the summer/fall decline in *N*. *ceranae* intensity is due to a decrease in daily temperature in these seasons [16, 49]. Moreover, more potent fat body development and an increase in the immune resistance before colony wintering in mid-latitudes [50] could also lead to a reduction in the *N*. *ceranae* infection intensity in the fall. This corresponds with the seasonal, permanent decrease in *N*. *ceranae* infection intensity presented in Fig 1. A dominant impact of temperature on the *N*. *ceranae* disease course was indirectly confirmed by a non-fluctuating pattern of the *N*. *ceranae* infection course in our cage tests carried out at the constant temperature of 30°C in which, after the initial decrease in *N*. *ceranae* spore intensity, their continuous rise was observed (Fig 3). This initial decrease resulted from the fact that *N*. *ceranae* needed about 3–4 days to complete its life cycle inside honey bee intestine cells [41, 51]. After that, newly formed spores were released into the midgut lumen and could be excreted with feces or they could invade next cells of the same host. Rapid acceleration in the *N*. *ceranae* infection intensity observed from the 8^th^ day of cage experiments (Fig 3) could be connected with a breakdown of honey bees’ immune defense and the beginning of *N*. *ceranae* invasion.

The European eastern range limit of *N*. *ceranae* was found in Ukraine [52], but there are no data about seasonal changes in the infection from this location. Therefore, data presented here show seasonality (with the additional mid-summer peak) of the *N*. *ceranae* infection intensity at the European eastern limit of its range for the first time.

A growth in the intestine yeast CFU number was observed when the intensity of the *N*. *ceranae* infection increased both in field and in cage tests (Table 2, Figs 3 and 4). This suggests that the *N*. *ceranae* infection created favorable conditions for the intestinal yeast growth. Intestinal yeast CFU numbers detected in cage tests were generally lower than those detected in field tests both in *N*. *ceranae*-infected and uninfected workers (Figs 1 and 4). These findings contradict studies by Gilliam *et al*. [26, 28, 29], in which caged honey bees had always more yeast than hive bees. In our research, we focused on the fungi belonging only to intestinal yeasts, such as *Candida* and *Saccharomyces* genera, whereas Gilliam *et al*. [26, 27, 28, 29] studied also other yeast genera. This might be the reason for this discrepancy suggesting that different intestinal yeast genera might respond to caged bees’ environment in different ways.

Generally, honey bees kept in fully functional colonies withstand a higher *Nosema* infection and pressure from greater yeast populations without any visible symptoms of colony weakening than the caged honey bees. Probably, a more varied diet, ability to fly, a stay in a fully functional colony, including presence of a queen bee, positively affected colony honey bees to deal with parasites and to gain appropriate overall fitness.

In mid-latitudes, foragers live under great pressure from May to the end of July when they have to collect supplies for the colony development and their whole energy demands are connected with flying and foraging. From August in the northern hemisphere, a honey bee colony switches to the overwintering preparation and workers consume more energy for maintaining immune defense than during spring or early summer [50]. Consequently, during late summer and fall, bees are well prepared to combat any microbial attack including fungal pathogens such as *Nosema* spp. or the opportunistic yeast pathogens such as *Candida* or *Saccharomyces*. This corresponds with our observation that the number of *N*. *ceranae* spores and intestinal yeast CFU number decreased in August. Two peaks in May and July were detected both for *N*. *ceranae* infection intensity and intestinal yeast CFU numbers (Figs 1 and 2). This additionally indicated that the reason for these rises lay probably in the seasonal differences in honey bee immune defense. The July peak may have been connected with the colony preparing to replace short-living summer honey bees with overwintering bees. Nevertheless, these data need further studies.

This natural seasonality in nosemosis development may be a cause of misunderstandings during experiments on new therapeutics against *N*. *ceranae*. A natural decrease in the number of *N*. *ceranae* spores during such experiments can be confounded with the effect of the *Nosema* spp. treatment with these new compounds. Therefore, we recommend performing all preliminary studies on new therapeutics in cages. Our investigation also showed that field studies on anti-*Nosema* drugs should be performed during two consecutive years, including colony overwintering, and only the heavily infected colonies threatened with extinction ought to be used. Colonies infected with *N*. *ceranae* at low intensity can be found naturally self-cured the following year.

Disturbances caused by *N*. *ceranae* infection lead to malabsorption of nutrients, which is *inter alia* manifested by the presence of large amounts of undigested sugars in feces of infected honey bees [53, 54]. This excess of sugar creates conditions favorable to rapid intestine yeast development. Consequently, sugar metabolism products in the growing yeast population increase acidity of the intestinal microenvironment. This, in turn, creates conditions favorable to *N*. *ceranae* development [55, 56, 57, 58] and a self-reinforcing circle begins. So, particularly in its initial stages, nosemosis may stimulate growth of intestinal yeast population, whereas an increase in the number of intestinal yeast, through feedback, may facilitate a development of nosemosis. However, during severe *N*. *ceranae* infection, a competition for nutrients and attachment sites begins [20]. When a honey bee’s intestine is filled with *Nosema* spores [24], yeast cells probably cannot convert into their *hypha* form and embed themselves into epithelial cells of the midgut. Moreover, *Nosema*-infected honey bees show decreased foraging abilities [59]. This is probably the cause of nutrient shortages, which are not suitable for yeast development. Consequently, *N*. *ceranae*, just like all intercellular parasites, draw energy directly from their host cells and may win the competition [20] with yeasts. At the end of our cage experiments, we observed a decrease in the yeast number (Fig 4), which suggests the onset of such competition. Consequently, during low and medium *Nosema* spp. infection intensity *N*. *ceranae* and yeasts positively affected their mutual development, however, at a high *Nosema* infection intensity, a suppressing effect of *Nosema* on yeast development was observed.

## Conclusion

In the European eastern limit of its range, *N*. *ceranae* infection intensity showed seasonality and was observed as a spring peak and subsequent decline in the summer and fall. However, we also observed an additional mid-summer peak that has not been noticed in other studies. Intestinal yeast population revealed a very similar seasonal pattern to that observed in *N*. *ceranae*. There is mutual interdependence between intestine yeast and *N*. *ceranae*. At a low and medium *Nosema* spp. intensity, both parasites positively affected their mutual development but at high infection intensity, a suppressive effect of *Nosema* on yeast development can be considered.

Due to seasonal changes in the *N*. *ceranae* infection intensity in honey bee colonies, we recommend performing studies on new anti-*Nosema* drugs during two consecutive years, including colony overwintering. A natural decrease in the number of *N*. *ceranae* spores during a season can be misinterpreted as an effect of a *Nosema* spp. treatment with new compounds.

Moreover, honey bee infection with *N*. *ceranae* supports additional opportunistic yeast infections, which may result in a faster colony depopulation.
